# Explainability, Bias and Generalizability of AI Models in Dentistry: A Systematic Review of Model Interpretability and Equity

**DOI:** 10.1002/cre2.70375

**Published:** 2026-05-15

**Authors:** Vini Mehta, Ankita Mathur, Mahati Bhadania, Cosimo Galletti, Javier Flores‐Fraile

**Affiliations:** ^1^ Dental Research Cell Dr. D. Y. Patil Dental College & Hospital, Dr. D. Y. Patil Vidyapeeth (Deemed to be University) Pune India; ^2^ Faculty of Dentistry University of Ibn al‐Nafis for Medical Sciences Sana'a Yemen; ^3^ London School of Hygiene and Tropical Medicine London UK; ^4^ School of Medicine and Surgery Kore University of Enna Enna Italy; ^5^ Department of Surgery University of Salamanca Salamanca Spain

**Keywords:** artificial intelligence, bias, dentistry, explainability accuracy, generalizability

## Abstract

**Background:**

AI‐based dentistry has advanced significantly in recent years. AI models like deep learning (DL) and machine learning (ML) have paved the way for new approaches to image diagnostics and early risk prediction, making patient treatment plans more personalized.

**Aim:**

The objective of this study was to assess the explainability, bias, and generalizability of AI models used in dentistry and evaluate the correlation between AI models.

**Methods:**

Four databases were searched to retrieve relevant research records. The protocol was registered with PROSPERO. The data extraction sheet was designed according to PRISMA guidelines, and the data were managed in MS Excel. Also, a correlation analysis was performed to determine the nature of the relationship between the variables using SPSS. All tests were performed at a 95% confidence interval. Additionally, a critical appraisal of the included studies was also performed using the PROBAST tool.

**Results:**

Eleven studies were included in this review. Overall, the assessment indicated variability in correlation strength between AI model accuracy and attributes of trustworthiness (*r* = 0.367–0.987). Analysis demonstrated the good performance of DL models (3D U‐Net; accuracy = 95.10%) relative to others (73%–98.20%). However, the heterogeneous nature of included studies (*n* = 11) focused on different dental domains like diagnosis, dental service use, and disease risk prediction, which limits its generalizability.

**Conclusion:**

Findings from this review indicated the importance of methodological rigor while using AI models in dentistry. Results suggest that the incorporation of trustworthiness attributes can improve dental treatment planning and early disease diagnosis.

## 1 Introduction

Artificial intelligence (AI) is increasingly used in dentistry to support clinicians in making informed decisions and treatment planning (Dhingra 2023). With its remarkable capabilities, AI has shown promising potential across many domains of dentistry, ranging from early disease risk prediction to personalized treatments (Kale et al. 2024). In addition, it has been leveraged to reduce human error in disease diagnosis and to streamline resource allocation, thereby enabling smoother workflows (Najeeb and Islam 2025). However, with the advancement in AI‐integrated dental care, there have been escalating perturbations about its interpretability and fairness (Koçak et al. 2024).

Along with growing concerns regarding the bias and explainability of AI models, dental clinicians also face an ethical dilemma in accepting AI‐based predictive outcomes (Agrawal et al. 2025). Studies have shown that when multiple conditions coexist in a single image, dentists would probably consider a set of possibilities rather than rely on a single AI prediction (Shi et al. 2024). This indicates that although mere findings regarding dental caries or periodontal bone loss are insufficient, limitations in identifying localized variations at the tooth level have led to concerns about the trustworthiness of AI models (Brima and Atemkeng 2024). For dental clinicians, this is especially significant as the acceptance and use of any AI tool largely depends on its results, as well as on a high level of trust in the generated outcomes (Naderalvojoud et al. 2025). Thus, the bias and generalizability of any AI model are key in ensuring equitable dental care (Chisini et al. 2025; Krois et al. 2021; Schuch et al. 2023). However, existing evidence indicates that an AI model's capacity to predict from its training data often involves trade‐offs (Chen et al. 2020). The risk of overfitting and the model's tendency to memorize the training data compromise overall performance on test datasets (Kawaguchi et al. 2017; Nay and Strandburg 2019; Röösli et al. 2022). In fact, many dentists also practice in decentralized settings, thereby increasing demographic variability and oral disease prevalence patterns (Norori et al. 2021). Therefore, considering similar aspects, chances of misalignment between clinical reasoning and algorithm predictions are consistent risk factors in implementing AI in different dental domains (Yuan et al. 2026).

Literature in this context indicates that AI trustworthiness also varies depending on the type of model used. For instance, the use of deep learning (DL) model architectures, such as convolutional neural networks, requires large and complex datasets to achieve high‐accuracy outcomes (Fassler et al. 2020; Kuwada et al. 2023). However, they are often regarded as “black boxes” due to their opaque decision‐making and the opaque methods used to arrive at a conclusion (Ahmad et al. 2021; Röösli et al. 2022). This can be a risky attribute in clinical decision‐making, leading to a clinician's dilemma of accepting or rejecting an outcome generated by AI models (Chinta et al. 2025; Cross et al. 2024). This inconclusive reasoning often leads to improper model explainability and limiting generalizability (Chakraborty et al. 2017; Koçak et al. 2024; Prajod et al. 2022). Likewise, machine learning (ML) models have shifted from traditional, explainable models to more complex ones. Model architecture made ML models more advanced, such as ensemble methods, using gradient boosting to combine multiple simple models into a single complex model. This has shifted explainable ML models more towards “black box” derivatives (Arsiwala‐Scheppach et al. 2023), making clinical decision‐making complex, hard to interpret, and limited in generalization for wider sections of communities (Kale et al. 2024; Shujaat 2025; Wang et al. 2025). This undermines the bias and equity of the AI model used in clinical decision‐making in different fields of dentistry. Therefore, this systematic review was undertaken to examine the qualitative aspects of AI models in dentistry, specifically explainability, bias, and generalizability, and to explore their relationship with model accuracy.

## 2 Materials and Methods

### 2.1 Protocol Registration

The study protocol was prospectively registered with the International Prospective Register of Systematic Reviews (PROSPERO; registration number: CRD20251171635). This systematic review was conducted and reported following the guidelines of the Preferred Reporting Items for Systematic Reviews and Meta‐Analyses (PRISMA) checklist (Page et al. 2021) (Table S1).

### 2.2 Focused Question

The systematic review aimed to answer the following research question: “Are AI‐based models in dentistry explainable, fair (free from bias), and/or generalizable? How does it relate to model accuracy?” This study employed the BeHEMoTh (Behavior of Interest, Health Context, Models or Theories) framework (Shujaat 2025), defined as follows (Hosseini et al. 2024):

- **Behavior of interest:** Explainability, bias, and/or generalizability of AI‐based models in dentistry and their relationship with model accuracy.
- **Health context:** Applications of AI within the field of dentistry.
- **Models/Theories:** Concepts of fairness and trustworthiness in AI‐driven dental diagnostics, disease risk prediction, or service delivery in dentistry.

### 2.3 Eligibility Criteria

To be eligible for inclusion in this systematic review, studies were required to:

a. Focus specifically on the explainability, bias, and/or generalizability of AI‐based dental models (diagnostic, disease risk prediction, dental health service optimization, or any other models that focus on oral health);
b. Employ any AI model (generative AI, DL, ML) as a main intervention.
c. Be primary research in its design, which includes retrospective or prospective modeling studies.
d. Be published in a peer‐reviewed journal.

Studies that did not meet these criteria were excluded. Additionally, preprints, book chapters, conference presentations, reviews, opinion pieces, commentaries, and dissertations were not considered.

### 2.4 Information Sources and Search Strategy

Two independent reviewers have searched the four electronic databases, PubMed/MEDLINE, Scopus, Embase, and Science Direct using Medical Subject Headings (MeSH) terms and title/abstract, with appropriate filters to narrow the search results. Boolean operators like “AND” and “OR” were also used to combine the different search terms in the search strategy (Table 1). A detailed search strategy is given in Table S2. Additionally, Google Scholar was manually searched for “gray literature.” Given the high results, only the first 100 pages were reviewed. Furthermore, references in the selected research studies were screened to identify additional potentially eligible studies.

**Table 1:** Search strategy for PubMed/MEDLINE.

Database	Search string
PubMed/MEDLINE	((((((“artificial intelligence”[MeSH* Terms]) AND (“dentistry”[MeSH Terms])) AND (“explainability”[Title/Abstract] OR “explainability accuracy”[Title/Abstract] OR “explainability ai”[Title/Abstract] OR “explainability algorithm”[Title/Abstract])) AND (“bias”[MeSH Terms])) OR (“bias”[Title/Abstract])) AND (“generalizability”[Title/Abstract])) AND (generalizability[MeSH Terms]); ((((“artificial intelligence”[MeSH Terms]) AND (“dentistry”[MeSH Terms])) AND (“explainability”[Title/Abstract] OR “explainability accuracy”[Title/Abstract]) AND (“bias”[MeSH Terms])) OR (“bias”[Title/Abstract])) AND (generalizability[MeSH Terms]); ((((“artificial intelligence”[MeSH Terms]) AND (“dentistry”[MeSH Terms])) AND (“explainability”[Title/Abstract]); ((((“artificial intelligence”[MeSH Terms]) AND (“dentistry”[MeSH Terms])) AND (“bias”[MeSH Terms])); ((((“artificial intelligence”[MeSH Terms]) AND (“dentistry”[MeSH Terms])) AND (generalizability[MeSH Terms])

* MeSH: medical subject headings.

### 2.5 Study Selection

Two independent reviewers imported data into the Rayyan AI Software. The pooled research records were screened through a multistage process. Initially, duplicates were removed from imported research records. Furthermore, the remaining articles were screened for title and abstract. After the first screening, selected articles were sought for full‐text retrieval. Finally, full‐text articles were screened, and only those that met the predefined eligibility criteria were included in the final review.

### 2.6 Data Extraction and Management

The systematic data extraction form was prepared using Microsoft Excel software (version 2402) in accordance with the PRISMA guidelines. The form covers important study characteristics, including study ID (authors/year), region, study design, AI model used, databases covered, AI algorithm type, overall sample size, outcome measured, and overall result. Furthermore, the quantitative data for the correlation analysis were also extracted using a separate data form for statistical analysis and to summarize the accuracy of each AI model. The data were curated as binary responses (yes/no) for each category of the outcome variable. The categorization of responses was made based on the information available in the included studies.

### 2.7 Data Items

Under this review, the following data items were considered for analyzing and formulating the result:

1. AI: It consists of algorithms that mimic human brain function, including advanced techniques such as ML and DL (Hosseini et al. 2024).
2. Explainability: It refers to the interpretability of AI models in the context of dentistry. This can be assessed using tools such as gradient‐weighted class activation mapping (Grad‐CAM) or Shapley Additive Explanations (SHAP), which are displayed as heatmaps in the studies (Brima and Atemkeng 2024).
3. Generalizability: It is the ability of AI models to validate their outcomes across a diverse set of data, regardless of geographical, ethnic, or gender variability. It was assessed based on the summaries or analyses provided in the included studies (Kawaguchi et al. 2017).
4. Bias: It refers to the extent of fairness in the applied AI models across different domains of dentistry. It was assessed based on the summaries or analyses provided in the included studies (Koçak et al. 2024).

### 2.8 Outcome Measures

The primary outcome of this study was to analyze the trustworthiness (qualitative characteristic) of AI‐based models in dentistry, focusing on explainability, bias, and/or generalizability. Furthermore, the secondary outcome of this systematic review was to analyze their relationship with model accuracy.

### 2.9 Data Synthesis

A confusion matrix was also prepared to summarize the performance of different AI models across various dental domains. Where necessary, narrative summaries were provided to enhance the comprehensibility and readability of the results.

#### 2.9.1 Correlation Matrix

Apart from the confusion matrix and study characteristics, a correlation analysis was also performed to produce a matrix to identify underlying patterns and the strength of the relationship between model characteristics. It must be noted that the correlation analysis performed was exploratory in nature and does not aim to establish any causal association between the variables. To perform this analysis, we have operationalized methodological characteristics into binary variables based on the work done by Murad et al. (2018), Liu et al. (2019), and Barredo Arrieta et al. (2020). This method was used to facilitate structured cross‐study comparison, as included studies were heterogeneous in nature and hence direct comparison would be difficult to perform. This allowed us to generate a consistent set of comparable indicators, which helped in identifying patterns in the truthfulness of AI models across heterogeneous studies. We pooled the performance of AI models in oral health and focused on descriptive indicators consisting of two aspects: functional interpretability and evidence evaluation dimensions. By functional interpretability dimension, mean features include grouping of conceptually equivalent categories based on model predictions, like feature attribution or attention‐based explanation. And by evidence‐based evaluation means focusing on an AI model's ability to ensure meaningful interpretation by human experts or support in clinical decision alignment. These descriptive indicators are features associated with explainability, generalizability or fairness/biasness in the model as reported by the included studies. These variables were operationalized as observable reporting indicators to enable structured cross‐study comparison. Variables were coded as “1” if the feature was descripted as presence, application, or narratively describing a positive effect. But if the description highlighted otherwise, then the variables were coded as “0.” This categorical classification for each variable was based on the common pattern among the included studies. However, correlation analysis was only performed to evaluate overall strength among the variables and model accuracy and does not indicate methodological equivalency among included studies. Also, it is important to appreciate that descriptive indicators were mainly reflecting the presence of methodological consideration within heterogeneous reporting contexts and do not intend to quantify the quality or effectiveness of the AI model implementation.

Furthermore, we understand that included studies may have used heterogeneous interpretability metrics like GRAD‐CAM, SHAP or LIME, each with distinct indicators, making it difficult for direct cross‐comparison. Statistical Package for the Social Sciences (SPSS, Version 27) was used to perform correlation analyses, with tests conducted at a 95% confidence interval to assess the relationship between explainability, bias, and/or generalizability and the accuracy of the AI models.

### 2.10 Risk of Bias Assessment

The Prediction model Risk of Bias Assessment Tool (PROBAST), which evaluates four main domains: participants, predictors, outcomes, and analysis, was used to assess the risk of bias (ROB) in the included model studies. This tool was proposed by Wolff et al. (2019). Two independent reviewers (A.M., M.B.) evaluated the ROB of the included studies. Each study was rated for risk of bias in the reported evidence as low, high, or unclear (Wolff et al. 2019). Furthermore, Cohen's kappa for the level of agreement between the independent authors was 0.92. Any conflict raised during the ROB assessment was resolved through mutual discussion and consensus with the third reviewer (V.M.).

## 3 Results

### 3.1 Study Selection

**Figure 1:**
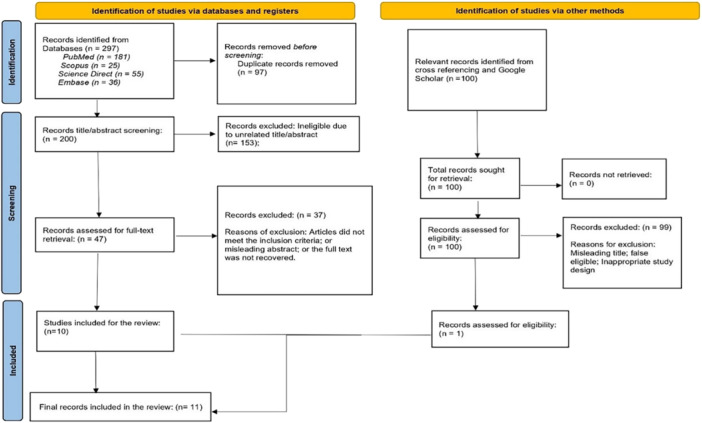
PRISMA flow diagram of study selection. Flowchart depicting the identification, screening, eligibility assessment, and final inclusion of studies in the systematic review of explainability, bias, and generalizability of AI models in dentistry.

A total of 297 research records were retrieved from four electronic databases: PubMed, Embase, Scopus, and ScienceDirect. Additionally, Google Scholar was used to identify relevant gray literature, yielding 75 records. After combining all sources, 97 duplicates were removed, leaving 200 records for title and abstract screening. Of these, 37 were deemed potentially eligible for full‐text review. Screening the references cited in these 37 articles identified 25 additional records for full‐text assessment. Following the complete screening process, 11 studies were ultimately included in the final review. Figure 1 illustrates the study selection process at each stage using a PRISMA flowchart. The excluded articles and the reasons for their exclusion are mentioned in Table S3.

### 3.2 Study Characteristics

Analysis of the included studies is represented in Table 2. All the studies were found to be retrospective modeling (*n* = 11) (Chisini et al. 2025; Erturk et al. 2025; Holtkamp et al. 2021; Krois et al. 2021; Long et al. 2024; Motmaen et al. 2024; Oztekin et al. 2023; Sreeram et al. 2025; Tirkkonen et al. 2025; Vinayahalingam et al. 2023). Many geographical regions were found to have been researched on this subject, including countries in Asia, Europe, and the USA. The overall sample size was 58,191 across 9 of the 11 included studies. However, one included study (Sreeram et al. 2025) did not clearly state the overall sample size. The majority of studies assessed model explainability, bias, and/or generalizability using deep learning (DL) models (*n* = 10), while Sreeram et al. (2025) and Tirkkonen et al. (2025) employed ML models. The DL models were largely unsupervised, whereas ML models were designed to be supervised. Notably, the majority of studies (*n* = 10) have assessed the outcomes of interest (explainability, bias, and/or generalizability of AI models) in the context of dental image diagnosis, except for two that focused on disease risk prediction (Tirkkonen et al. 2025) and dental service use prediction in Southern Brazil (Chisini et al. 2025). This analysis indicates that although AI models were extensively used in dentistry, particularly for image diagnostics to support clinical decision‐making, studies did not comprehensively address explainability, bias, and generalizability. A detailed BeHEMoTh framework for included studies was given in Table S4.

**Table 2:** Study characteristics.

Study ID	Region	Study design	Overall sample size	AI model used	Algorithm type	Outcome reported	Overall results
Krois et al. (2021)	Germany	Retrospective cross‐sectional	650	DL^a^	Unsupervised learning	Generalizability and explainability of the AI^b^ model in dental image analysis	The models did not show generalizability and explainability of AI model in dental image analysis
Holtkamp et al. (2021)	Switzerland	Retrospective cross‐sectional	226	DL	Unsupervised learning	Generalizability of deep learning models for caries detection in near‐infrared light transillumination images	The development DL models should be critically appraised for their generalizability and accuracy
Oztekin et al. (2023)	Turkey	Modeling study	13870	DL	Unsupervised learning	Explainability of AI model	Improved interpretability of the dental caries using DL dental model
Vinayahalingam et al. (2023)	Netherlands	Modeling study	162	DL	Unsupervised learning	Validation of AI model in an automated segmentation tool based on a DL algorithm for accurate 3D reconstruction of TMJ^d^	Although it demonstrated validity of the DL model in 3D reconstruction of TMJ, study demonstrated limited robustness and generalizability are risks
Motmaen et al. (2024)	Germany	Retrospective cross‐sectional	26,956	DL	Unsupervised learning	Explainability of AI model for predicting the need for tooth extraction from PAN^c^	AI performance improves with increasing contextual information and outperforms dentists/specialists in predicting tooth extraction
Long et al. (2024)	China	Single‐center retrospective study	372	DL	Unsupervised learning	Explainability of AI model for predicting the probability of success in DPC^e^	Model interpretability had supported decision‐making process for ensuring of success in DPC
Tirkkonen et al. (2025)	Finland	Modeling study	9686	ML^f^	Supervised learning	Complexity of interpreting of the ML model's risk prediction	Performance of our ML model during external validation degraded notably compared to the internal validation
Sreeram et al. (2025)	India	Retrospective modeling	Not mentioned clearly	DL	Unsupervised learning	Assessment of reliability of AI‐assisted diagnoses of dental radiographs and interpretable DL models in dentistry.	AI‐assisted diagnoses of dental radiographs and interpretable DL models in dentistry
Pan et al. (2025)	China	Modeling study	1056	DL	Unsupervised learning	Explainability, generalizability and bias of the CNN^g^ models for automatic mandibular canal localization on multicenter CBCT^h^ images	External validation and interpretability have shown significant improvements in ensuring clinical application potential
Chisini et al. (2025)	Brazil	Modeling study	3461	ML	Supervised learning	Focused on fairness (bias) of the ML models in predicting the use of dental services among adults aged 18 and older.	Performance of ML model varied across age and gender especially in case of mixed‐race individuals.
Erturk et al. (2025)	Turkey	Retrospective modeling study	1752	DL	Unsupervised learning	Focused on explainability approach to explain deep CNN models for automatic staging of periodontal bone loss severity using bite‐wing radiographs	YOLOv8 (deep CNN model) was successful in staging periodontal bone loss severity using bite‐wing radiographs with explainability approach

^a^ DL: deep learning.

^b^ AI: artificial intelligence.

^c^ PAN: panoramic radiographs.

^d^ TMJ: temporo‐mandibular joint.

^e^ DPC: direct pulp capping.

^f^ ML: machine learning.

^g^ CNN: convolutional neural network.

^h^ CBCT: cone beam computed tomography.

### 3.3 Correlation Between the AI Model Accuracy and Attributes of Trustworthiness

The relationship between AI models used in different fields of dentistry, and their trustworthiness (explainability, bias, and/or generalizability) is analyzed using a correlation matrix (Table 3). Overall, the analysis revealed that the strength and direction of correlation between AI model accuracies and trustworthiness characteristics are variable (Table 3). Explainability demonstrated a stronger positive correlation with AI model accuracy (*r* = 0.729; *p* < 0.013) than the other two attributes. Bias in the AI models showed a strong to moderate positive correlation with model accuracy (*r* = 0.663–0.782). Similarly, AI model accuracy and generalizability analyses indicated a weak to moderate positive correlation (*r* = 0.367–0.674). This suggests that although generalizability may not affect AI model accuracy statistically, components of external validity determine its performance on testing datasets.

**Table 3:** Correlation analysis between outcome variables and the accuracy of the different AI models.

Variables		Explainability (*r*; *p*‐value)	Bias/Fairness of AI^a^ models (*r*; *p*‐value)			Generalizability (*r*; *p*‐value)		Accuracy of AI models (*r*; *p*‐value)
			Dataset diversity	Subgroup analysis	Equity/Bias discussed	External validation	Robustness testing	
Explainability		1.00	0.918 (< 0.001)	0.966 (< 0.001)	0.927 (< 0.001)	0.873 (< 0.001)	0.870 (< 0.001)	**0.729 (0.013)**
Bias/Fairness of AI models	Dataset diversity	0.918 (< 0.001)	1.00	0.926 (< 0.001)	0.982 (< 0.001)	0.928 (< 0.001)	0.894 (< 0.001)	0.782 (0.189)
	Subgroup analysis	0.966 (< 0.001)	0.926 (< 0.001)	1.00	0.943 (< 0.001)	0.922 (< 0.001)	0.895 (< 0.001)	0.638 (0.294)
	Socio‐demographic equity	0.927 (< 0.001)	0.982 (< 0.001)	0.926 (< 0.001)	1.00	0.922 (< 0.001)	0.895 (< 0.001)	0.663 (0.152)
Generalizability	External validation	0.873 (< 0.001)	0.928 (< 0.001)	0.922 (< 0.001)	0.922 (< 0.001)	1.00	0.987 (< 0.001)	**0.367 (0.002)**
	Robustness testing	0.870 (< 0.001)	0.894 (< 0.001)	0.895 (< 0.001)	0.895 (< 0.001)	0.987 (< 0.001)	1.00	0.674 (0.183)
Accuracy of AI models		0.729 (0.013)	0.782 (0.189)	0.638 (0.294)	0.663 (0.152)	0.367 (0.335)	0.674 (0.183)	1.00

*Note:* Yellow‐highlighted cells represent diagonal elements (*r* = 1.00), indicating self‐correlation of variables, Bold values denote significance.

^a^ AI: artificial intelligence.

### 3.4 Explainability

Results from Table 3 highlight that a strong positive correlation was found between explainability and bias and the generalizability of the model (*r* = 0.729–0.966; *p* < 0.001). Analysis also revealed that the explainability of these AI models was moderately correlated with overall model accuracy, suggesting that higher model interpretability contributes to the overall model accuracy. Additionally, upon evaluation of the studies, it was found that they largely used CAMERAS, Grad‐CAM, or SHAP values as common tools to evaluate explainability characteristics.

### 3.5 Bias

The fairness of AI models across different dental areas, in terms of internal consistency and model accuracy, is reported in Table 3. The analysis revealed that data diversity, subgroup analysis, and socio‐demographic equity are strongly correlated with each other (*r* = 0.926–0.982; *p* < 0.001) as well as the model's explainability and generalizability (*r* = 0.870–0.966; *p* < 0.001). However, when bias in AI models was evaluated in relation to model accuracy, a moderate correlation was observed (*r* = 0.663–0.728, *p* > 0.05).

### 3.6 Generalizability

The correlation between model generalizability and other outcome measures, and between model accuracy and other outcome measures, is presented in Table 3. Across the included studies that evaluated generalizability, the robustness of the models was found to be strongly and positively correlated with bias and explainability (or interpretability) (*r* = 0.870–0.987; *p* < 0.001), indicating a clear interdependence among these factors. Additionally, external validation of the models showed a positive but weak correlation with accuracy, suggesting that model accuracy may be influenced by its external validity.

### 3.7 Performance of Different AI Models

The accuracy of different AI models across the included studies is reported in Table 4. Overall, the analysis indicates that DL models (3D U‐Net) outperform ML models (XGBoost, CatBoost Classifier, Gradient Boosting Classifier, Artificial Neural Network) for diagnosis, dental service use, and disease risk prediction.

**Table 4:** AI‐model performance matrix.

Study ID	AI model	Model sub‐classification	Accuracy	Sensitivity	Specificity	F1 score
Krois et al. (2021)	DL^a^	CNN^b^	64%	50.90%	99.93%	42.70%
Holtkamp et al. (2021)	DL	CNN	78%	76.0%	79.0%	73.0%
Oztekin et al. (2023)	DL	EfficientNet‐B0	90%	83.0%	97.0%	89.25%
		DenseNet	91.83%	87.33%	96.33%	91.45%
		ResNet 50	92%	87.33%	96.67%	91.61%
Vinayahalingam et al. (2023)	DL	3D U‐net	98.9%–99.5%	96.1%–97.8%	97.4%–97.5%	96.6%–97.6%
Motmaen et al. (2024)	DL	ResNet50	83.40%	79.90%	84.30%	66.10%
Long et al. (2024)	DL	DT^c^	69%	89.00%	25.00%	89.00%
		SVM^d^	82%	87.00%	50.00%	88.00%
		LR^e^	79%	72.00%	25.00%	76.00%
		RF^f^	83%	87.00%	50.00%	88.00%
		KNN^g^	74%	76.00%	50.00%	82.00%
Tirkkonen et al. (2025)	ML^h^	XGBoost^i^	82.10%	42.00%	91.60%	51.60%
Sreeram et al. (2025)	DL	U‐Net	97%–98%	79.40%	84.20%	73.90%
Pan et al. (2025)	DL	3D U‐net	86.8%	N/A^j^	N/A	N/A
Chisini et al. (2025)	ML	CatBoost classifier model	73%	83%	73%	73%
		Gradient boosting classifier	76%	76%	77%	77%
		Artificial neural network	73%	83%	73%	73%
Erturk et al. (2025)	DL	DCNN^k^	83.61%	82.48%	81.24%	81.55%

^a^ DL: deep learning.

^b^ CNN: convolutional neural network.

^c^ DT: decision tree.

^d^ SVM: support vector machine.

^e^ LR: logistic regression.

^f^ RF: random forest.

^g^ KNN: Kernel nearest neighbors.

^h^ ML: machine learning.

^i^ XGBoost: eXtreme gradient boost.

^j^ N/A: not available.

^k^ DCNN: deep convolutional neural network.

#### 3.7.1 Deep Learning Models

The included studies reported different levels of DL model accuracy (Table 4). Of the 11 included studies, 10 focused on the performance of different DL models in dental image diagnosis and dental service use (Arsiwala‐Scheppach et al. 2023; Chisini et al. 2025; Erturk et al. 2025; Holtkamp et al. 2021; Long et al. 2024; Motmaen et al. 2024; Oztekin et al. 2023; Sreeram et al. 2025; Vinayahalingam et al. 2023). A comparison of different DL models showed that 3D U‐Net, DenseNet, and ResNet50 performed comparatively better. However, models generally perform better on predefined, familiar datasets compared to external, novel datasets, with performance ranging from 51.0% to 77.2%. Notably, in a study, it was found that 3D U‐Net performance was not compromised even when the testing dataset was anonymous (95.10%). This suggests that the 3D U‐Net model structure supports not only interpretability but also minimizes bias while ensuring maximum generalizability. However, this may be contextually relevant to the models' defined objective and architecture. Additionally, analysis revealed that the majority of the models were validated using either 10‐fold or 5‐fold cross‐validation or binary cross‐entropy loss, indicating robustness in model validation beyond the training datasets.

### 3.8 Machine Learning Models

ML model's performance was also reported in terms of measured accuracy (Table 4). Only 2 of the 11 included studies (Chisini et al. 2025; Tirkkonen et al. 2025) used an ML model (XGBoost, CatBoost Classifier model, Gradient Boosting Classifier, Artificial Neural Network) to demonstrate its explainability and generalizability. The study assessed the model's accuracy in predicting dental caries risk and dental service use among adults in Southern Brazil. Analysis revealed that all the ML models perform better with a predefined dataset (accuracy = 73%–98.20%) than with an anonymous dataset. This model used a 10 and 5‐fold cross‐validation tool to assess its applicability across different settings. One of the included studies focused on explainability variable 35, and the other focused on dataset bias and variation in model output. This indicates that the generalizability of ML models was not clearly evaluated.

### 3.9 Quality Assessment

**Figure 2:**
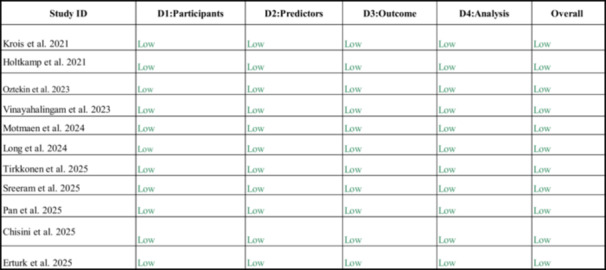
Risk of bias assessment using PROBAST. Summary of risk of bias and applicability concerns for each included prediction model study, evaluated across the four PROBAST domains: participants, predictors, outcome, and analysis.

The quality assessment of the included studies evaluated their methodological rigor and the strength of the supporting evidence. Overall, the critical appraisal of the 11 studies indicated a low risk of bias in the reported outcomes when analyzed using the PROBAST tool (Figure 2).

## 4 Discussion

This systematic review examined the explainability, bias, and generalizability of applied AI in dentistry. The study found a strong, positive relationship between these variables, which may or may not affect the overall accuracy of the AI model. Furthermore, it was also found that AI models that performed better in terms of explainability, bias and generalizability were often found within the same dental investigation type, such as diagnostic imaging. Therefore, exploratory consequences of the results were reflected rather than a causal relationship between these attributes. However, this can be ascribed to contextual differences in the tasks they perform, which often involve manual feature engineering of structured data, compared to DL models. Shujaat (2025) reported similar observations, where ML required expert oversight for preprocessing and regular maintenance, thus limiting its performance in dental imaging.

The correlation analysis revealed that AI models were more strongly related to each other when compared on explainability, bias, and/or generalizability than on accuracy. Accuracy appeared to be relatively independent of these model characteristics. This suggests that while the qualitative features of AI models are interdependent, they do not necessarily influence model performance, which reflects a quantitative characteristic. This observation was made by Ferrara (2023), highlighting that the robustness of AI models is affected by their algorithmic structure and the quality of the dataset rather than their qualitative characteristics. However, the study recognized the importance of dataset diversity to make it more robust and generalizable. A similar observation was also made by the Qamar and Bawany (2023) study, which reported a strong positive intervariable correlation, suggesting that all three outcome variables complemented one another. For instance, if a model is interpretable and bias‐free, its generalization performance also increases. This observation was further supported by the findings of Arsiwala‐Scheppach et al. (2023), who discussed the importance of interpretable ML models in dentistry and suggested that robust calibration and evaluation of the models have an important role in their generalization. However, in a study by Ennab and Mcheick (2022), it was concluded that model success increases when it is explainable, bias‐free and suitable for diverse datasets, underscoring the importance of robust data in healthcare and its relation with model accuracy. Therefore, this can be an important factor in ensuring fair and equitable decision‐making in dental image diagnosis and treatment planning. Furthermore, results from the correlation analysis indicated that the accuracy of these models is largely unaffected by robustness, except for explainability (data diversity) and generalizability (external validation). This can be attributed to the diverse datasets that ensure model reliability and minimal bias. This observation was given by Vimbi et al. (2024) and Garouani et al. (2025), indicating that if the AI models are more interpretable, their consistency and generalizability increase. Therefore, making AI models more interpretable enhances their explainability in dentistry, ensuring robustness in innovation and the validation of externally developed systems, and ensuring equity in dental health services.

Additionally, our review revealed that DL models performed more accurately in complex tasks, such as analyzing and predicting outcomes from dental radiographs. Similar observations were made by Dhingra (2023), indicating that DL model performance was more sophisticated than that of other AI models, although accuracy depended on the objective of the task. In fact, observations reported by Huang et al. (2022), Arsiwala‐Scheppach et al. (2023), Dey et al. (2024), and Tan et al. (2025) concluded that DL models possess the ability to autonomously extract complex features from input datasets, leading to more refined outputs, particularly in the case of convolutional neural CNNs when compared with traditional ML models. However, this does not mean ML models are less explainable. The findings in this review can be attributed to the heterogeneous study type and dataset characteristics contributing to the observed performance difference between AI models. It can be inferred from this observation that different domains in dentistry may demonstrate different performances when different AI models are used, and hence may not represent a competitive overview regarding this aspect. Traditional ML models are self‐explanatory and interpretable, acting as a “white box.” Similarly, Doshi‐Velez and Kim et al. (2017) and Guidotti et al. (2018) also highlight that traditional ML models like linear regression and decision trees were “inherently interpretable” and do not require post‐hoc explanations. Interestingly, AI model subcategories revealed that 3D U‐Net, DenseNet, and ResNet50 performed better, demonstrating superior capability for interpreting datasets with greater robustness, fairness, and generalizability. This can be attributed to the ability of these models, which enables them to integrate deep hierarchical learning features, precise segmentation of input data, advanced connectivity (skip and dense links), deep neural network functioning, and layered processing of raw data with strengthened gradient flow at each step, ensuring robustness of the models. This observation was supported by Lin et al. (2023), Rezaie et al. (2024), Mehta et al. (2024), and Kot et al. (2025), suggesting that DL models may perform more accurately (Schneider 1971). Petkovic (2023) has argued in one of the articles that for an AI model to be trustworthy, accuracy and explainability must be considered together, not as a trade‐off between accuracy and explainability.

Methodological robustness of the included studies suggested high confidence in the reported outcomes. In fact, this review also identified interesting asymmetries and limitations in the evidence presented by the included studies regarding the application of AI models in dentistry. The most concerning domain in which such studies were conducted was dental radiographic image diagnostics. Although numerous studies demonstrated the impact AI has on dentistry, the contextual considerations of explainability, bias, and generalizability for these advanced models were very limited. Furthermore, studies have shown limited attention to cross‐country dataset integration when examining AI model characteristics and applications in dentistry. Therefore, future research can focus on evaluating the integration of explainability features like comprehensive GRAD‐CAM heat maps with AI models like 3D U‐Net, and their role in mitigating “automation bias.” Following this, researchers can also evaluate interpretability performance by comparing two different groups, such as dental students and experienced dental clinicians, using dental radiographs, paving the way in implementation science in dentistry.

### 4.1 Strengths and Limitations

This systematic review used both statistical analysis and narrative review to evaluate the trustworthiness (explainability, bias, and generalizability) of different AI models and their relationship with model performance, providing a holistic view of AI models behavior with the data. This study is the first of its kind to use structured statistical analysis to examine the interdependence between qualitative (explainability, bias, generalizability) and quantitative model characteristics (model performance), providing insights beyond the technical aspects of AI models in dentistry. Nonetheless, this study also contributes to the responsible and ethical use of AI by emphasizing the importance of AI model trustworthiness, especially in healthcare.

However, this review has also been subject to various limitations. The study was unable to present a holistic picture of other domains of dental health sciences, as most of the evidence was available in radiographic image diagnostics. Although our review reflects on the importance of AI model trustworthiness in field dentistry, observed results should be interpreted cautiously as the number of studies was relatively small. This limits the generalizability of the study's overall findings. Furthermore, correlation analysis was performed based on qualitative characteristics of the AI models where interpretation of the authors regarding any of the AI models from the included studies could have led to subjective bias. Binary characterization of the complex features of AI models, such as explainability and fairness, may have resulted in limited inferential strengths. This indicates that findings can only be considered exploratory rather than confirmatory. Another key limitation of this study was the heterogeneous nature of the available evidence. Additionally, the absence of a standardized tool for assessing bias and generalizability further restricted the applicability of the findings. Since a quantitative data synthesis through meta‐analysis could not be performed, the inability to estimate potential publication bias represents an additional limitation of this study.

## 5 Conclusion

This review highlighted the importance of AI model trustworthiness in dentistry, particularly with respect to explainability, bias, and generalizability and emerging patterns of methodologies followed while incorporating AI in dental clinical settings. Although AI models used in dentistry may demonstrate improved interpretability, fairness, and generalizability, these qualities do not always correspond to higher accuracy. These findings largely remain preliminary due to study heterogeneity, restricted reporting of AI‐model functioning in dentistry, limited sample size and exploratory correlation analysis based on binary coding. Nonetheless, explainability and external data validation were found to influence model performance in real‐world applications. Therefore, this review emphasizes the need to balance fairness, generalizability, and interpretability with performance optimization to ensure that dental AI systems are both accurate and clinically meaningful, maximizing the benefits of emerging technologies for improved human health outcomes.

## Author Contributions

Conceptualization: Vini Mehta, Ankita Mathur, and Mahati Bhadania. Methodology: Vini Mehta, Ankita Mathur, and Mahati Bhadania. Data curation: Vini Mehta, Ankita Mathur, and Mahati Bhadania. Formal analysis: Vini Mehta, Ankita Mathur, and Mahati Bhadania. Investigation (material preparation, data collection): Vini Mehta, Ankita Mathur, and Mahati Bhadania. Writing – original draft: Ankita Mathur, Mahati Bhadania, and Cosimo Galletti. Writing – review and editing: Ankita Mathur, Mahati Bhadania, Cosimo Galletti, Vini Mehta, and Javier Flores‐Fraile. Visualization (figure preparation): Cosimo Galletti and Javier Flores‐Fraile. Supervision: Vini Mehta and Ankita Mathur. Project administration: Vini Mehta and Ankita Mathur.

## Funding

The authors have nothing to report.

## Ethics Statement

This systematic review was conducted and reported following the guidelines of the Preferred Reporting Items for Systematic Reviews and Meta‐Analyses (PRISMA) checklist. The study protocol was prospectively registered with the International Prospective Register of Systematic Reviews (PROSPERO; registration number: CRD20251171635).

## Consent

The authors have nothing to report.

## Conflicts of Interest

The authors declare no conflicts of interest.

## Supporting information


**Table S1:** PRISMA Checklist.
**Table S2:** Elaborated search strategy.
**Table S3:** List of excluded articles.
**Table S4:** BeHEMoTh Framework for included studies.

## Data Availability

The data that supports the findings of this study are available in the Supporting Information of this article.
