# Supplementary material for: Explainability, Bias and Generalizability of AI Models in Dentistry: A Systematic Review of Model Interpretability and Equity
Source: Clin Exp Dent Res. 2026 May 15;12(3):e70375. doi: 10.1002/cre2.70375 (PMC13178149; doi:10.1002/cre2.70375)
Supplement: Supplementary file 1 — Table S1: PRISMA Checklist. Table S2: Elaborated search strategy. Table S3: List of excluded articles. Table S4: BeHEMoTh Framework for included studies. [file CRE2-12-e70375-s001.docx]

**SUPPLEMENTARY MATERIAL**

**Supplementary Table 1: PRISMA Checklist**

| **Section and Topic** | **Item #** | **Checklist item** | **Location where item is reported** |
| --- | --- | --- | --- |
| **TITLE** | | |  |
| Title | 1 | Identify the report as a systematic review. | Page 01 |
| **ABSTRACT** | | |  |
| Abstract | 2 | See the PRISMA 2020 for Abstracts checklist. | Page 01-02 |
| **INTRODUCTION** | | |  |
| Rationale | 3 | Describe the rationale for the review in the context of existing knowledge. | Page 03-04 |
| Objectives | 4 | Provide an explicit statement of the objective(s) or question(s) the review addresses. | Page 04 |
| **METHODS** | | |  |
| Eligibility criteria | 5 | Specify the inclusion and exclusion criteria for the review and how studies were grouped for the syntheses. | Page 04-05 |
| Information sources | 6 | Specify all databases, registers, websites, organisations, reference lists and other sources searched or consulted to identify studies. Specify the date when each source was last searched or consulted. | Page 05 |
| Search strategy | 7 | Present the full search strategies for all databases, registers and websites, including any filters and limits used. | Page 05-06; Supplementary document= Page 01 |
| Selection process | 8 | Specify the methods used to decide whether a study met the inclusion criteria of the review, including how many reviewers screened each record and each report retrieved, whether they worked independently, and if applicable, details of automation tools used in the process. | Page 06 |
| Data collection process | 9 | Specify the methods used to collect data from reports, including how many reviewers collected data from each report, whether they worked independently, any processes for obtaining or confirming data from study investigators, and if applicable, details of automation tools used in the process. | Page 06 |
| Data items | 10a | List and define all outcomes for which data were sought. Specify whether all results that were compatible with each outcome domain in each study were sought (e.g. for all measures, time points, analyses), and if not, the methods used to decide which results to collect. | Page 06-07 |
|  | 10b | List and define all other variables for which data were sought (e.g. participant and intervention characteristics, funding sources). Describe any assumptions made about any missing or unclear information. | Page 06-07 |
| Study risk of bias assessment | 11 | Specify the methods used to assess risk of bias in the included studies, including details of the tool(s) used, how many reviewers assessed each study and whether they worked independently, and if applicable, details of automation tools used in the process. | Page 07 |
| Effect measures | 12 | Specify for each outcome the effect measure(s) (e.g. risk ratio, mean difference) used in the synthesis or presentation of results. | Page 07 |
| Synthesis methods | 13a | Describe the processes used to decide which studies were eligible for each synthesis (e.g. tabulating the study intervention characteristics and comparing against the planned groups for each synthesis (item #5)). | Page 07 |
|  | 13b | Describe any methods required to prepare the data for presentation or synthesis, such as handling of missing summary statistics, or data conversions. | Page 07 |
|  | 13c | Describe any methods used to tabulate or visually display results of individual studies and syntheses. | Page 07 |
|  | 13d | Describe any methods used to synthesize results and provide a rationale for the choice(s). If meta-analysis was performed, describe the model(s), method(s) to identify the presence and extent of statistical heterogeneity, and software package(s) used. | Not Applicable |
|  | 13e | Describe any methods used to explore possible causes of heterogeneity among study results (e.g. subgroup analysis, meta-regression). | Not Applicable |
|  | 13f | Describe any sensitivity analyses conducted to assess robustness of the synthesized results. | Not Applicable |
| Reporting bias assessment | 14 | Describe any methods used to assess risk of bias due to missing results in a synthesis (arising from reporting biases). | Not Applicable |
| Certainty assessment | 15 | Describe any methods used to assess certainty (or confidence) in the body of evidence for an outcome. | Not Applicable |
| **RESULTS** | | |  |
| Study selection | 16a | Describe the results of the search and selection process, from the number of records identified in the search to the number of studies included in the review, ideally using a flow diagram. | Page 08 |
|  | 16b | Cite studies that might appear to meet the inclusion criteria, but which were excluded, and explain why they were excluded. | Page 08 |
| Study characteristics | 17 | Cite each included study and present its characteristics. | Page 09-12 |
| Risk of bias in studies | 18 | Present assessments of risk of bias for each included study. | Page 17-18 |
| Results of individual studies | 19 | For all outcomes, present, for each study: (a) summary statistics for each group (where appropriate) and (b) an effect estimate and its precision (e.g. confidence/credible interval), ideally using structured tables or plots. | Page 13-17 |
| Results of syntheses | 20a | For each synthesis, briefly summarise the characteristics and risk of bias among contributing studies. | Page 18 |
|  | 20b | Present results of all statistical syntheses conducted. If meta-analysis was done, present for each the summary estimate and its precision (e.g. confidence/credible interval) and measures of statistical heterogeneity. If comparing groups, describe the direction of the effect. | Not Applicable |
|  | 20c | Present results of all investigations of possible causes of heterogeneity among study results. | Not Applicable |
|  | 20d | Present results of all sensitivity analyses conducted to assess the robustness of the synthesized results. | Not Applicable |
| Reporting biases | 21 | Present assessments of risk of bias due to missing results (arising from reporting biases) for each synthesis assessed. | Not Applicable |
| Certainty of evidence | 22 | Present assessments of certainty (or confidence) in the body of evidence for each outcome assessed. | Not Applicable |
| **DISCUSSION** | | |  |
| Discussion | 23a | Provide a general interpretation of the results in the context of other evidence. | Page 19-21 |
|  | 23b | Discuss any limitations of the evidence included in the review. | Page 21 |
|  | 23c | Discuss any limitations of the review processes used. | Page 21 |
|  | 23d | Discuss implications of the results for practice, policy, and future research. | Page 21 |
| **OTHER INFORMATION** | | |  |
| Registration and protocol | 24a | Provide registration information for the review, including register name and registration number, or state that the review was not registered. | Page 04 |
|  | 24b | Indicate where the review protocol can be accessed, or state that a protocol was not prepared. | Page 04 |
|  | 24c | Describe and explain any amendments to information provided at registration or in the protocol. | Not applicable |
| Support | 25 | Describe sources of financial or non-financial support for the review, and the role of the funders or sponsors in the review. | Title Page |
| Competing interests | 26 | Declare any competing interests of review authors. | Title Page |
| Availability of data, code and other materials | 27 | Report which of the following are publicly available and where they can be found: template data collection forms; data extracted from included studies; data used for all analyses; analytic code; any other materials used in the review. | Title Page |

**Supplementary Table 2: Elaborated search strategy**

| **Databases** | **Search Strategy** |
| --- | --- |
| Scopus | ( TITLE-ABS-KEY ( artificial intelligence ) AND TITLE-ABS-KEY ( explainability ) OR TITLE-ABS-KEY ( bias ) OR TITLE-ABS-KEY ( generalizability ) AND TITLE-ABS-KEY ( oral health ) ); ( TITLE-ABS-KEY ( artificial intelligence ) AND TITLE-ABS-KEY ( explainability ) OR TITLE-ABS-KEY ( bias ) OR TITLE-ABS-KEY ( generalizability ) AND TITLE-ABS-KEY ( endodontics ) OR TITLE-ABS-KEY ( periodontal disease ) OR TITLE-ABS-KEY ( oral AND maxillofacial surgery ) AND TITLE-ABS-KEY ( prosthodontics ) OR TITLE-ABS-KEY ( oral pathology ) OR TITLE-ABS-KEY ( dental image diagnosis ) ); ( TITLE-ABS-KEY ( artificial intelligence ) AND TITLE-ABS-KEY ( dentistry ) AND TITLE-ABS-KEY ( explainability ) OR TITLE-ABS-KEY ( bias ) OR TITLE-ABS-KEY ( generalizability ) ) |
| Embase | ('interpretability'/exp OR 'interpretability' OR 'generalizability':ti,ab,kw OR 'algorithm bias':ti,ab,kw) AND 'artificial intelligence-assisted technology':ti,ab,kw AND 'dentistry':ti,ab,kw; ('dentistry'/exp OR 'dentistry') AND 'artificial intelligence':ti,ab,kw AND generalisability:ti,ab,kw AND 'explainability':ti,ab,kw OR 'algorithm bias':ti,ab,kw |
| Science Direct | ('generalizability' OR 'explainable machine learning' OR 'explainable artificial intelligence' OR 'algorithm bias') AND 'dentistry' |

**Supplementary Table 3:** **List of excluded articles**

| **Study Title** | **Author and Year** | **Reason for exclusion** |
| --- | --- | --- |
| Performance of a neural network trained to make third-molar treatment-planning decisions (1) | Brickley M.R. and Shepherd J.P 1996 | This article has misleading title under name neural networks and does not directly focus on the explainability, bias and generalizability of AI model application in dentistry |
| A new mathematical modelling based shape extraction technique for Forensic Odontology (2) | Jaffino et al. 2017 | Full-text review of this article does not meet the “*Behaviour of Interest”* criteria of the study |
| Accuracy of dental identification of individuals with unrestored permanent teeth by visual comparison with radiographs of mixed dentition (3) | Gorza L and Mânica S 2018 | This article does not directly focus on the explainability, bias and generalizability of AI model application in dentistry |
| Dentnet: Deep neural transfer network for the detection of periodontal bone loss using panoramic dental radiographs (4) | Kim et al. 2019 | This article does not directly focus on the explainability, bias and generalizability of AI model application in dentistry |
| Automatic human identification from panoramic dental radiographs using the convolutional neural network (5) | Fan et al. 2020 | This article does not directly focus on the explainability, bias and generalizability of AI model application in dentistry |
| Panoramic Dental Radiography Image Enhancement Using Multiscale Mathematical Morphology (6) | Román et al. 2021 | Full-text review of this article does not meet the “*Behaviour of Interest and Health Context”* criteria of the study |
| Deep learning based diagnosis for cysts and tumors of jaw with massive healthy samples (7) | Yu et al. 2022 | Full-text review of this article does not meet the “*Behaviour of Interest and Health Context”* criteria of the study |
| Fairness of Machine Learning Algorithms for Predicting Foregone Preventive Dental Care for Adults (8) | Schuch et al. 2023 | Full-text review of this article does not meet the “*Behaviour of Interest”* criteria of the study |
| Assessment of the quality of different commercial providers using artificial intelligence for automated cephalometric analysis compared to human orthodontic experts (9) | Kunz et al. 2023 | This article does not directly focus on the explainability, bias and generalizability of AI model application in dentistry |
| Positional assessment of lower third molar and mandibular canal using explainable artificial intelligence (10) | Kempers et al. 2023 | This article does not directly focus on the explainability, bias and generalizability of AI model application in dentistry |
| Artificial intelligence (A.I.) in dental curricula: Ethics and responsible integration (11) | Kim et al. 2023 | This article does not meet the inclusion criteria |
| Predicting dental caries outcomes in young adults using machine learning approach (12) | Ogwo et al. 2023 | This article does has not been peer reviewed yet |
| Deep learning-based segmentation of dental implants on cone-beam computed tomography images: A validation study (13) | Elgarba et al. 2023 | This article does not directly focus on the explainability, bias and generalizability of AI model application in dentistry |
| Exploring the Practical Applications of Artificial Intelligence, Deep Learning, and Machine Learning in Maxillofacial Surgery: A Comprehensive Analysis of Published Works (14) | Czako et al. 2024 | Full-text review of this article does not meet the “*Behaviour of Interest”* criteria of the study |
| DentalSegmentator: Robust open source deep learning-based CT and CBCT image segmentation (15) | Dot et al. 2024 | Full-text review of this article does not meet the “*Behaviour of Interest”* criteria of the study |
| Performance evaluation of three versions of a convolutional neural network for object detection and segmentation using a multiclass and reduced panoramic radiograph dataset (16) | Bonfanti-Gris et al. 2024 | Full-text review of this article does not meet the “*Behaviour of Interest”* criteria of the study |
| Harnessing artificial intelligence role in oral cancer diagnosis and prediction: A comprehensive exploration (17) | Behara et al. 2024 | Full-text screening of the article does not meet the inclusion criteria |
| Enhanced Diagnostic Accuracy for Dental Caries and Anomalies in Panoramic Radiographs Using a Custom Deep Learning Model (18) | Bhat et al. 2024 | This article does not directly focus on the explainability, bias and generalizability of AI model application in dentistry |
| Identifying predictors of the tooth loss phenotype in a large periodontitis patient cohort using a machine learning approach (19) | Lee et al. 2024 | This article does not directly focus on the explainability, bias and generalizability of AI model application in dentistry |
| Detection of periodontal bone loss patterns and furcation defects from panoramic radiographs using deep learning algorithm: a retrospective study (20) | Kurt-Bayrakdar et al. 2024 | This article does not directly focus on the explainability, bias and generalizability of AI model application in dentistry |
| Deep Learning-Based Detection of Separated Root Canal Instruments in Panoramic Radiographs Using a U (2)-Net Architecture (21) | İnönü et al. 2025 | This article does not meet the inclusion criteria |
| Detection of Fractured Endodontic Instruments in Periapical Radiographs: A Comparative Study of YOLOv8 and Mask R-CNN (22) | Çetinkaya et al. 2025 | This article does not directly focus on the explainability, bias and generalizability of AI model application in dentistry |
| An AI-assisted explainable mTMCNN architecture for detection of mandibular third molar presence from panoramic radiography (23) | Kayadibi et al. 2025 | This article does not meet the inclusion criteria |
| Unsupervised tooth segmentation from three dimensional scans of the dental arch using domain adaptation of synthetic data (24) | Arian et al. 2025 | This article does not directly focus on the explainability, bias and generalizability of AI model application in dentistry |
| Evaluating the accuracy of generative artificial intelligence models in dental age estimation based on the Demirjian's method (25) | Abuabara et al. 2025 | This article does not directly focus on the explainability, bias and generalizability of AI model application in dentistry |
| AI Efficiency in Dentistry: Comparing Artificial Intelligence Systems with Human Practitioners in Assessing Several Periodontal Parameters (26) | Butnaru et al. 2025 | This article does not meet the inclusion criteria |
| Explainable screening of oral cancer via deep learning and case-based reasoning (27) | Cimino et al. 2025 | This article does not meet the inclusion criteria |
| AI-driven approaches in the management of early childhood caries: A path toward global oral health (28) | Nayak et al. 2025 | This article does not directly focus on the explainability, bias and generalizability of AI model application in dentistry |
| A deep learning approach based on YOLO v11 for automatic detection of jaw cysts (29) | Kaygisiz et al. 2025 | This article does not directly focus on the explainability, bias and generalizability of AI model application in dentistry |
| A hybrid convolutional neural network model for dental age estimation using buccal alveolar bone level for Saudi children (30) | Nezhad et al. 2025 | This article does not directly focus on the explainability, bias and generalizability of AI model application in dentistry |
| Diagnostic accuracy of an artificial intelligence-based software in detecting supernumerary and congenitally missing teeth in panoramic radiographs (31) | Makrygiannakis et al. 2025 | This article does not directly focus on the explainability, bias and generalizability of AI model application in dentistry |
| Charting New Territory: AI Applications in Dental Caries Detection from Panoramic Imaging (32) | Hung et al. 2025 | This article does not directly focus on the explainability, bias and generalizability of AI model application in dentistry |
| Revolutionizing the diagnosis of dental caries using artificial intelligence-based methods (33) | Marwaha et al. 2025 | This article does not meet the inclusion criteria |
| An Explainable Deep Learning Framework for Mandibular Canal Segmentation from Cone Beam Computed Tomography Volumes (34) | Barzas et al. 2025 | This article does not directly focus meet the inclusion criteria as it is a conference paper. |
| A Comparison of Deep Learning vs. Dental Implantologists in Cone-Beam Computed Tomography-Based Bone Quality Classification (35) | Pornvoranant et al. 2025 | This article does not directly focus on the explainability, bias and generalizability of AI model application in dentistry |
| DenPAR: Annotated Intra-Oral Periapical Radiographs Dataset for Machine Learning (36) | Rasnayaka et al. 2025 | This article does not directly focus on the explainability, bias and generalizability of AI model application in dentistry |
| E-dentification, the use of teledentistry for remote personal forensic identification in forensic odontology: a Queensland experience (37) | Evans et al. 2025 | Full-text review of this article does not meet the “*Behaviour of Interest and Health Context”* criteria of the study |

**Supplementary Table 4: BeHEMoTh Framework for included studies**

| **Study ID** | **Behaviour of Interest** | **Health Context** | **Models/Theories** |
| --- | --- | --- | --- |
| Krois et al. 2021 (38) | Generalizability and explainability of the AI^1^ | Dental image analysis | DL^2^ |
| Holtkamp et al. 2021 (39) | Generalizability of AI model | Dental image analysis | DL |
| Oztekin et al. 2023 (40) | Explainability of AI model | Dental caries diagnosis | DL |
| Motmaen et al. 2024 (41) | Explainability of AI model | predicting the need for tooth extraction from PAN^3^ | DL |
| Vinayahalingam et al. 2023 (42) | Validation (generalizability, bias) of AI model in an automated segmentation tool | Demonstrated validity of the DL model in 3D reconstruction of TMJ^4^ | DL |
| Long et al. 2024 (43) | Explainability of AI model | Predicting probability of success in DPC^5^ | DL |
| Tirkkonen et al. 2025 (44) | Validation (generalizability, bias) of AI model | Dental disease risk prediction | ML^6^ |
| Sreeram et al. 2025 (45) | Assessment of reliability of AI-assisted diagnoses of dental radiographs and interpretable DL models in dentistry. | AI-assisted diagnoses of dental radiographs | DL |
| Pan et al. 2025 (46) | Explainability, generalizability and bias of the CNN^7^ models | External validation and interpretability of automatic mandibular canal localization on multicenter CBCT^8^ images | DL |
| Chisini et al. 2025 (47) | Focused on fairness (bias) of the ML models | Predicting the use of dental services among adults aged 18 and older | ML |
| Erturk et al. 2025 (48) | Focused on explainability approach to explain deep CNN models | YOLOv8 (deep CNN model) was used for automatic staging of periodontal bone loss severity using bite-wing radiographs | DL |

1: AI: Artificial intelligence; 2: DL: Deep learning; 3: PAN: Panoramic radiographs; 4: TMJ: Temporo-mandibular joint; 5: DPC: Direct pulp capping;

6: ML: Machine learning; 7: CNN: Convolutional neural network; 8: CBCT: Cone Beam Computed Tomography

1: DL: Deep learning; 2: AI: Artificial intelligence; 3: PAN: Panoramic radiographs; 4: TMJ: Temporo-mandibular joint; 5: DPC: Direct pulp capping; 6: ML: Machine learning; 7: CNN: Convolutional neural network; 8: CBCT: Cone Beam Computed Tomography

1: DL: Deep learning; 2: AI: Artificial intelligence; 3: PAN: Panoramic radiographs; 4: TMJ: Temporo-mandibular joint; 5: DPC: Direct pulp capping; 6: ML: Machine learning; 7: CNN: Convolutional neural network; 8: CBCT: Cone Beam Computed Tomography

**References**

1. Brickley MR, Shepherd JP. Performance of a Neural Network Trained to Make Third-molar Treatment-planning Decisions. Med Decis Making. 1996 Jun;16(2):153–60.

2. G. J, A. B, Gurunathan U, B. V, J. PJ. A new mathematical modelling based shape extraction technique for Forensic Odontology. Journal of Forensic and Legal Medicine. 2017 Apr;47:39–45.

3. Gorza L, Mânica S. Accuracy of dental identification of individuals with unrestored permanent teeth by visual comparison with radiographs of mixed dentition. Forensic Science International. 2018 Aug;289:337–43.

4. Kim J, Lee HS, Song IS, Jung KH. DeNTNet: Deep Neural Transfer Network for the detection of periodontal bone loss using panoramic dental radiographs. Sci Rep. 2019 Nov 26;9(1):17615.

5. Fan F, Ke W, Wu W, Tian X, Lyu T, Liu Y, et al. Automatic human identification from panoramic dental radiographs using the convolutional neural network. Forensic Science International. 2020 Sep;314:110416.

6. Román JCM, Fretes VR, Adorno CG, Silva RG, Noguera JLV, Legal-Ayala H, et al. Panoramic Dental Radiography Image Enhancement Using Multiscale Mathematical Morphology. Sensors. 2021 Apr 29;21(9):3110.

7. Yu D, Hu J, Feng Z, Song M, Zhu H. Deep learning based diagnosis for cysts and tumors of jaw with massive healthy samples. Sci Rep. 2022 Feb 3;12(1):1855.

8. Schuch HS, Furtado M, Silva GFDS, Kawachi I, Chiavegatto Filho ADP, Elani HW. Fairness of Machine Learning Algorithms for Predicting Foregone Preventive Dental Care for Adults. JAMA Netw Open. 2023 Nov 3;6(11):e2341625.

9. Kunz F, Stellzig-Eisenhauer A, Widmaier LM, Zeman F, Boldt J. Assessment of the quality of different commercial providers using artificial intelligence for automated cephalometric analysis compared to human orthodontic experts. J Orofac Orthop. 2025 May;86(3):145–60.

10. Kempers S, Van Lierop P, Hsu TMH, Moin DA, Bergé S, Ghaeminia H, et al. Positional assessment of lower third molar and mandibular canal using explainable artificial intelligence. Journal of Dentistry. 2023 Jun;133:104519.

11. Kim CS, Samaniego CS, Sousa Melo SL, Brachvogel WA, Baskaran K, Rulli D. Artificial intelligence (A.I.) in dental curricula: Ethics and responsible integration. Journal of Dental Education. 2023 Nov;87(11):1570–3.

12. Ogwo C, Grant B, Warren J, Caplan D, Levy S. Predicting Dental Caries Outcomes in Young Adults Using Machine Learning Approach [Internet]. 2023 [cited 2025 Nov 6]. Available from: https://www.researchsquare.com/article/rs-3393538/v1

13. Elgarba BM, Van Aelst S, Swaity A, Morgan N, Shujaat S, Jacobs R. Deep learning-based segmentation of dental implants on cone-beam computed tomography images: A validation study. Journal of Dentistry. 2023 Oct;137:104639.

14. Czako L, Sufliarsky B, Simko K, Sovis M, Vidova I, Farska J, et al. Exploring the Practical Applications of Artificial Intelligence, Deep Learning, and Machine Learning in Maxillofacial Surgery: A Comprehensive Analysis of Published Works. Bioengineering. 2024 Jul 3;11(7):679.

15. Dot G, Chaurasia A, Dubois G, Savoldelli C, Haghighat S, Azimian S, et al. DentalSegmentator: Robust open source deep learning-based CT and CBCT image segmentation. Journal of Dentistry. 2024 Aug;147:105130.

16. Bonfanti-Gris M, Ruales E, Salido M, Martinez-Rus F, Özcan M, Pradies G. Artificial intelligence for dental implant classification and peri-implant pathology identification in 2D radiographs: A systematic review. Journal of Dentistry. 2025 Feb;153:105533.

17. Behera A, Babu NA, Renuka RR, Dharmalingam Jothinathan MK. Harnessing artificial intelligence role in oral cancer diagnosis and prediction: A comprehensive exploration. Oral Oncology Reports. 2024 Jun;10:100314.

18. Bhat S, Birajdar G, Patil M. Enhanced Diagnostic Accuracy for Dental Caries and Anomalies in Panoramic Radiographs Using a Custom Deep Learning Model. Cureus [Internet]. 2024 Aug 20 [cited 2025 Nov 6]; Available from: https://www.cureus.com/articles/284611-enhanced-diagnostic-accuracy-for-dental-caries-and-anomalies-in-panoramic-radiographs-using-a-custom-deep-learning-model

19. Lee CT, Zhang K, Li W, Tang K, Ling Y, Walji MF, et al. Identifying predictors of the tooth loss phenotype in a large periodontitis patient cohort using a machine learning approach. Journal of Dentistry. 2024 May;144:104921.

20. Kurt-Bayrakdar S, Bayrakdar İŞ, Yavuz MB, Sali N, Çelik Ö, Köse O, et al. Detection of periodontal bone loss patterns and furcation defects from panoramic radiographs using deep learning algorithm: a retrospective study. BMC Oral Health. 2024 Jan 31;24(1):155.

21. İnönü N, Aksoy U, Kırmızı D, Aksoy S, Akkaya N, Orhan K. Deep Learning-Based Detection of Separated Root Canal Instruments in Panoramic Radiographs Using a U2-Net Architecture. 2025;

22. Çetinkaya İ, Çatmabacak ED, Öztürk E. Detection of Fractured Endodontic Instruments in Periapical Radiographs: A Comparative Study of YOLOv8 and Mask R-CNN. Diagnostics. 2025 Mar 7;15(6):653.

23. Kayadibi İ, Köse U, Güraksın GE, Çetin B. An AI-assisted explainable mTMCNN architecture for detection of mandibular third molar presence from panoramic radiography. International Journal of Medical Informatics. 2025 Mar;195:105724.

24. Arian MSH, Sifat FA, Ahmed S, Mohammed N, Farook TH. Unsupervised tooth segmentation from three dimensional scans of the dental arch using domain adaptation of synthetic data. International Journal of Medical Informatics. 2025 Mar;195:105769.

25. Abuabara A, Do Nascimento TVPM, Trentini SM, Costa Gonçalves AM, Hueb De Menezes-Oliveira MA, Madalena IR, et al. Evaluating the accuracy of generative artificial intelligence models in dental age estimation based on the Demirjian’s method. Front Dent Med. 2025 Jul 29;6:1634006.

26. Butnaru OM, Tatarciuc M, Luchian I, Tudorici T, Balcos C, Budala DG, et al. AI Efficiency in Dentistry: Comparing Artificial Intelligence Systems with Human Practitioners in Assessing Several Periodontal Parameters. Medicina. 2025 Mar 23;61(4):572.

27. Cimino MGCA, Campisi G, Galatolo FA, Neri P, Tozzo P, Parola M, et al. Explainable screening of oral cancer via deep learning and case-based reasoning. Smart Health. 2025 Mar;35:100538.

28. Nayak PP, Shetty V, S S, Zacharias L, Gore I. AI-driven approaches in the management of early childhood caries: A path toward global oral health. Journal of Oral Biology and Craniofacial Research. 2025 Sep;15(5):1134–40.

29. Kaygısız ÖF, Uranbey Ö, Gürsoytrak B, Gür ZB, Çiçek A, Canbal MA. A deep learning approach based on YOLO v11 for automatic detection of jaw cysts. BMC Oral Health. 2025 Oct 2;25(1):1518.

30. Nezhad SMM, Bakhsh HH, Ishtiaq U, Rahmat RAA, ElKhateeb SM. A hybrid convolutional neural network model for dental age estimation using buccal alveolar bone level for Saudi children.

31. Makrygiannakis MA, Giannakopoulos K, Kavadella A, Paraskevis D, Kaklamanos EG. Diagnostic accuracy of an artificial intelligence-based software in detecting supernumerary and congenitally missing teeth in panoramic radiographs. European Journal of Orthodontics. 2025 Jun 12;47(4):cjaf054.

32. Hung M, Yevseyevich D, Khazana M, Schwartz C, Lipsky MS. Charting New Territory: AI Applications in Dental Caries Detection from Panoramic Imaging. Dentistry Journal. 2025 Aug 12;13(8):366.

33. Marwaha J, Singla M, Nath A, Arya A. Revolutionizing the diagnosis of dental caries using artificial intelligence-based methods. Journal of Conservative Dentistry and Endodontics. 2025 May;28(5):401–5.

34. Barzas K, Fouad S, Jasa G, Landini G. An Explainable Deep Learning Framework for Mandibular Canal Segmentation from Cone Beam Computed Tomography Volumes. In: Bansal MS, Chen W, Khudyakov Y, Măndoiu II, Moussa MR, Patterson M, et al., editors. Computational Advances in Bio and Medical Sciences [Internet]. Cham: Springer Nature Switzerland; 2025 [cited 2025 Nov 6]. p. 1–13. (Lecture Notes in Computer Science; vol. 14548). Available from: https://link.springer.com/10.1007/978-3-031-82768-6_1

35. Pornvoranant T, Panyarak W, Wantanajittikul K, Charuakkra A, Rungsiyakull P, Chaijareenont P. A Comparison of Deep Learning vs. Dental Implantologists in Cone-Beam Computed Tomography-Based Bone Quality Classification. J Digit Imaging Inform med. 2024 Nov 18;38(4):2462–71.

36. Rasnayaka S, Leuke Bandara D, Jayasundara A, Jayasinghe R, Wimalasiri C, Rathnayake P, et al. DenPAR: Annotated Intra-Oral Periapical Radiographs Dataset for Machine Learning. Sci Data. 2025 Oct 3;12(1):1615.

37. Evans N, Soon A, Forrest A, Meredith M, Harris P. E-dentification, the use of teledentistry for remote personal forensic identification in forensic odontology: a Queensland experience. Forensic Sciences Research. 2025 Jun 20;10(3):owaf016.

38. Krois J, Garcia Cantu A, Chaurasia A, Patil R, Chaudhari PK, Gaudin R, et al. Generalizability of deep learning models for dental image analysis. Sci Rep. 2021 Mar 17;11(1):6102.

39. Holtkamp A, Elhennawy K, Cejudo Grano De Oro JE, Krois J, Paris S, Schwendicke F. Generalizability of Deep Learning Models for Caries Detection in Near-Infrared Light Transillumination Images. JCM. 2021 Mar 1;10(5):961.

40. Oztekin F, Katar O, Sadak F, Yildirim M, Cakar H, Aydogan M, et al. An Explainable Deep Learning Model to Prediction Dental Caries Using Panoramic Radiograph Images. Diagnostics. 2023 Jan 7;13(2):226.

41. Motmaen I, Xie K, Schönbrunn L, Berens J, Grunert K, Plum AM, et al. Insights into Predicting Tooth Extraction from Panoramic Dental Images: Artificial Intelligence vs. Dentists. Clin Oral Invest. 2024 Jun 18;28(7):381.

42. Vinayahalingam S, Berends B, Baan F, Moin DA, Van Luijn R, Bergé S, et al. Deep learning for automated segmentation of the temporomandibular joint. Journal of Dentistry. 2023 May;132:104475.

43. Long Y, Xu X, Chen J, Liu S, Li J, Dong Y. An explainable predictive model of direct pulp capping in carious mature permanent teeth. Journal of Dentistry. 2024 Oct;149:105269.

44. Tirkkonen O, Tiensuu H, Väyrynen E, Suutala J, Ville V, Laitala ML, et al. An Explainable and Transparent Machine Learning Approach for Predicting Dental Caries: A Cross-National Validation Study [Internet]. 2025 [cited 2025 Oct 16]. Available from: https://www.researchsquare.com/article/rs-6783190/v1

45. Sreeram A, R. B, Mn. A. Explainable AI for Panoramic Dental Radiographs Using Contrastive Learning and U-Net Based Segmentation. JSCP. 2025 Jun;7(2):114–23.

46. Pan X, Wang C, Luo X, Dong Q, Sun H, Zhang W, et al. Development and verification of a convolutional neural network-based model for automatic mandibular canal localization on multicenter CBCT images. BMC Oral Health. 2025 Aug 21;25(1):1352.

47. Chisini LA, Araújo CF, Delpino FM, Figueiredo LM, Filho ADPC, Schuch HS, et al. Dental services use prediction among adults in Southern Brazil: A gender and racial fairness-oriented machine learning approach. Journal of Dentistry. 2025 Oct;161:105929.

48. Erturk M, Öziç MÜ, Tassoker M. Deep Convolutional Neural Network for Automated Staging of Periodontal Bone Loss Severity on Bite-wing Radiographs: An Eigen-CAM Explainability Mapping Approach. J Digit Imaging Inform med. 2024 Aug 15;38(1):556–75.
